# Overweight and its associated factors among employees of a university from the state of Santa Catarina

**DOI:** 10.47626/1679-4435-2020-533

**Published:** 2020-12-11

**Authors:** Fernanda de Oliveira Meller, Antonio José Grande, Micaela Rabelo Quadra, Antônio Augusto Schäfer

**Affiliations:** 1 Programa de Pós-Graduação em Saúde Coletiva, Universidade do Extremo Sul Catarinense - Criciúma (SC), Brazil; 2 Curso de Medicina, Universidade Estadual de Mato Grosso do Sul - Campo Grande (MS), Brazil

**Keywords:** overweight, obesity, risk factors, worker’s health, cross-sectional studies

## Abstract

**Introduction:**

The increasing incidence of overweight worldwide is influenced by several factors of daily life and also affects the working population.

**Objective:**

To assess overweight and its association with sociodemographic factors, food consumption, and eating habits in employees of a university.

**Method:**

This is a cross-sectional study conducted with employees of a university in southern Santa Catarina. A questionnaire containing demographic, socioeconomic, and nutritional information was used. The exposure variables studied were: sex, age, marital status, schooling, frequency of weekly food consumption, and eating behaviors. Overweight was assessed using body mass index. Crude and adjusted analyses of the association between overweight and independent variables were performed using Poisson’s regression.

**Results:**

The prevalence of overweight among the 214 employees was 54.9%. After the adjusted analysis, women had a 34% lower risk of overweight when compared to men (prevalence ratio: 0.66; 95% confidence interval 0.53-0.82). In addition, overweight was directly associated with age (p <0.001), while schooling remained inversely associated with overweight.

**Conclusions:**

The high prevalence of overweight among workers demonstrates the need to develop interventions and /or programs that promote health in the work environment, especially for groups at higher risk, such as older men and those with lower levels of schooling.

## INTRODUCTION

Overweight is defined as the accumulation of excessive fat in body tissues, causing adverse health effects such as morbidity and mortality.^1^ Together with other factors, such as systemic arterial hypertension, increased glycemia, smoking, and a sedentary lifestyle, overweight is responsible for increasing the risk of cardiovascular diseases, cancer, type 2 diabetes mellitus, osteoarthritis, among other chronic non-communicable diseases.^2^

Worldwide, the prevalence of overweight among adults was 39% in 2014.^3^ In Brazil, for 8 years, the prevalence increased 13.4%, and is currently 53.7% (57.7 and 50.5% among men and women, respectively).^4^ The situation is even more worrying when analyzing the prevalence in the southern region of the country, where 67.5% of adults are overweight (67.6% in the state of Santa Catarina).^5^

The factors that may be related to this high prevalence are the food transformations that have occurred in recent years all over the world, in which the consumption of natural foods has been declining and the consumption of processed foods has been growing very significantly, which leads to unbalanced food consumption in nutrients and energy.^6^ In addition, the decrease in homemade meals, the increase in fast food consumption, and the short periods for eating meals are consequences of urbanization that are also related to the increase in overweight.^7^

According to some studies, the workers with the highest rates of obesity are men, older, with low schooling, married, and those who eat fat and do not have access to fruit in the workplace.^8^^,^^9^ Few existing studies assess the presence of overweight in workers; however, identifying the nutritional status of this population to create strategies to combat overweight is highly relevant.^9^ Thus, to contribute to the expansion of studies in this area, this study aimed to assess overweight and its associated factors in employees of a university in southern Santa Catarina.

## METHOD

This study was approved by the Research Ethics Committee of the institution, under approval number 59682816.3.0000.0119. All workers who agreed to participate in the research signed a free and informed consent form, containing all information related to the research.

This is a cross-sectional study conducted from 2016 to 2017 with employees of a university located in the south of the state of Santa Catarina. The sample size was calculated considering the total number of employees, the prevalence of the result, and the 95% confidence interval (95%CI). Next, 15% was added for losses and refusals, totaling 267 individuals to be interviewed. A proportional sampling by sector of work of the institution (n = 19) was performed, and then, the employees were drawn by simple sampling.

For data collection, trained interviewers administered a semi-structured questionnaire covering demographic, socioeconomic, and nutritional information, in addition to questions related to eating habits and food consumption to all employees who agreed to participate in the study.

To assess both food consumption and eating behavior, the questionnaire administered was the same used for monitoring risk and protective factors for chronic diseases by telephone survey (Vigilância de Fatores de Risco e Proteção para Doenças Crônicas por Inquérito Telefônico -Vigitel).^4^ Information was collected on the weekly consumption of the following foods, considering the consumption of the last week before the interview: vegetables, fruit, legumes, meat, soft drinks, artificial juices, and sweets. The response alternatives were “never”, “seldom”, “1 to 2 days”, “3 to 4 days”, “5 to 6 days” or “daily”. The variable consumption was dichotomized in “< 5 days a week” and “≥ 5 days a week”. As for eating behavior, the following were included: the number of meals per day (<3, 4, ≥ 5), the place where meals are eaten (home, work, restaurant), the habit of eating while watching television (no or yes), the habit of consuming the visible fat on red meat (no or yes) and on chicken meat (no or yes), and the habit of adding salt to the food after cooking (no or yes).

Regarding the outcome variable “overweight”, the body mass index (BMI) was calculated using the self-reported weight and height of the workers. Individuals with BMI values up to 24.9 kg/m^2^, were classified as “without overweight” and those with BMI values greater than 24.9 kg/m^2^, as “overweight”.

Sociodemographic variables were also studied: sex (men or women), age (18-27, 28-37, 38-47, 48-60 years), marital status (single, married, separated / divorced / widowed) and years of schooling (0-8, 9-11, 12 years or longer). Descriptive analyses of qualitative variables showing absolute (n) and relative (%) frequencies were performed, besides its respective 95%CIs.

**Figura 1:**
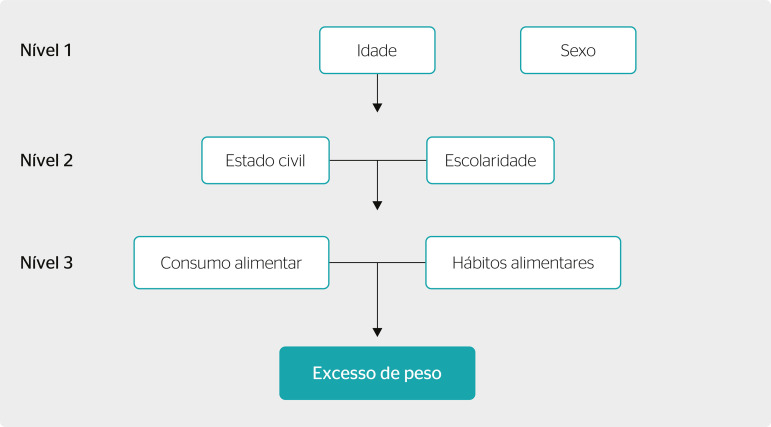
Hierarchical model of the factors associated with overweight of the employees studied.

The crude and adjusted analyses of the association between overweight and the independent variables were performed using Poisson’s regression, showing the p-value corresponding to the Wald test for heterogeneity or linear trend for ordinal categorical variables. For the adjusted analysis, we used the hierarchical model presented in Figure 1 and the backward method for the inclusion of variables. All exposure variables were part of the analysis, and those with a p-value lower than 0.20 remained as possible confounding factors.

The analyses were performed using Stata, version 12.1 (StataCorp LLC, Texas, USA).

## RESULTS

Of the total of 267 employees selected to participate in the study, there were 53 losses and/or refusals, totaling 214 employees studied.

Table 1 shows the characteristics of the workers. We verified that most of them were women (65.3%; 95%CI 58.8-71.7) aged between 18 and 37 years (66.4%; 95%CI 60.0-72.7). We also observed that approximately one-half of the sample was single (44.4%; 95%CI 37.7-51.1) and had 12 years or more of schooling (47.6%; 95%CI 40.8-54.4). The prevalence of overweight among employees was 54.9% (95%CI 48.1-61.8).

**Table 1:** Characteristics of employees of a university in the municipality of Criciúma, state of Santa Catarina, 2016/2017 (n = 214).

Variables	n (%)	95%CI
Sex		
Men	74 (34.7)	283-41.2
Women	139 (65.3)	58.8-71.7
Age (years)		
18-27	70 (32.7)	26.4-39.0
28-37	72 (33.6)	27.3-40.0
38-47	39 (18.2)	13.0-23.4
48-60	33 (15.5)	10.5-20.3
Marital status		
Single	95 (44.4)	37.7-51.1
Married	99 (46.3)	39.5-53.0
Separated/divorced/widowed	20 (9.3)	5.4-13.3
Schooling (in complete years)*		
0-8	24 (11.4)	7.1-15.8
9-11	86 (41.0)	34.2-47.7
12 or more	100 (47.6)	40.8-54.4
Overweight		
No	92 (45.1)	38.2-52.0
Yes	112 (54.9)	48.1-61.8

95%CI: 95% confidence interval

* Maximum percentage of unknown observations for the schooling variable: 1.9% (n = 4): 1.9% (n = 4).

Table 2 shows the weekly food consumption and eating habits of the employees studied. We observed that about one-half of them consumed fruit and vegetables less than 5 days a week (51.9%; 95%CI 45.1-58.6 and 44.4%; 95%CI 37.7-51, 1, respectively). Few employees reported consuming legumes 5 days a week or more (37.4%; 95%CI 30.8-43.9). Also, about one-fifth of the sample consumed soft drinks / artificial juices and sweets at least 5 days a week (15.4%; 95%CI 10.5-20.3 and 22.9%; 95%CI 17.2 -28.6, respectively).

**Table 2:** Frequency of weekly consumption and eating habits of employees of a university in the municipality of Criciúma, state of Santa Catarina, 2016/2017 (n = 214). (n = 214).

Variables	n (%)	95%CI
Legumes		
< 5 days	134 (62.6)	56.1-69.2
5 days or more	80 (37.4)	30.8-43.9
Vegetables/Legumes		
< 5 days	95 (44.4)	37.7-51.1
5 days or more	119 (55.6)	48.9-62.3
Fruit		
< 5 days	111 (51.9)	45,1-58,6
5 days or more	103 (48.1)	41,4-54,9
Red meat		
< 5 days	171 (79.9)	74.5-85.3
5 days or more	43 (20.1)	14.7-25.5
Chicken meat		
< 5 days	172 (80.4)	75.0-85.7
5 days or more	42 (19.6)	14.3-25.0
Soft drinks/ artificial juices		
< 5 days	101 (84.6)	79.7-89.5
5 days or more	33 (15.4)	10.5-20.3
Sweets		
< 5 days	165 (77.1)	71.4-82.8
5 days or more	49 (22.9)	17.2-28.6
Number of meals per day		
< 3	44 (20.7)	15.2-26.1
4	77 (36.2)	29.6-42.7
> 5	92 (43.1)	36.5-49.9
Place of meals		
Home	95 (45.0)	38.3-51.8
Work	102 (48.4)	41.5-55.1
Restaurant	14 (6.6)	3.2-10.0
Habit of eating while watching TV		
No	111 (52.9)	46.1-59.7
Yes	99 (47.1)	40.3-53.9
Consumption of the visible fat on red meat*		
No	145 (74.4)	68.2-80.5
Yes	50 (25.6)	19.5-31.8
Consumption of the visible fat on chicken meat*		
No	155 (74.5)	68.5-80.5
Yes	53 (25.5)	19.5-31.5
Habit of adding salt to food after cooking		
No	194 (90.6)	86.7-94.6
Yes	20 (9.4)	5.4-13.3

95%CI: 95% confidence interval

* Maximum percentage of unknown observations for the variable consumption of the visible fat on red meat: 8.9% (n = 19).

Approximately one-half of the employees had 5 or more meals a day (43.1%; 95%CI 36.5-49.9), had their meals at work (48.4%; 95%CI 41.5-55.1), and had the habit of eating while watching television (47.1%; 95%CI 40.3-53.9). Also, we observed that one-quarter of the workers consumed the visible fat on red meat and on chicken meat (25.6%; 95%CI 19.5-31.8 and 25.5%, respectively) (Table 2).

The crude and adjusted analyses of the association between overweight and the independent variables studied are shown in Tables 3 and 4, respectively. We verified that, even after adjusting for possible confounding factors, women had a 34% lower risk of being overweight when compared to men (PR: 0.66; 95%CI 0.53-0.82). The age variable was directly associated with being overweight, that is, the older the age, the greater the probability of overweight, even after adjusted analysis (p < 0.001). It can also be observed that, after adjustment, overweight remained inversely associated with schooling. Employees with 12 years or more of study had a 27% lower risk of being overweight when compared to those with up to 8 years of schooling (p = 0.050).

**Table 3:** Crude analysis of the association between overweight and the independent variables of employees at a university in the municipality of Criciúma, state of Santa Catarina, 2016/2017 (n = 214).

Variables	n	RP (95%CI)	P
Sex			0.001*
Men	52	1	
Women	60	0.66 (0.52-0.84)	
Age			< 0.001*
18-27	22	1	
28-37	38	1.68 (1.12-2.51)	
38-47	26	2.08 (1.39-3.13)	
48-60	26	2.64 (1.82-3.83)	
Marital status			0.092*
Single	42	1	
Married	58	1.35 (1.03-1.78)	
Separated/divorced/widower	12	1.30 (0.85-1.98)	
Schooling (years)			0.002^†^
0-8	16	1	
9-11	46	0.67 (0.51-0.88)	
12 or more	47	0.56 (0.42-0.75)	
Consumption of legumes (weekly)			0.219*
< 5 days	65	1	
5 days or more	47	1.17 (0.91-1.50)	
Consumption de vegetables/legumes (weekly)			0.929*
< 5 days	48	1	
5 days or more	64	1.01 (0.79-1.30)	
Consumption of fruit (weekly)			0.510*
< 5 days	60	1	
5 days or more	52	0.92 (0.72-1.18)	
Consumption of red meat (weekly)			0.860*
< 5 days	90	1	
5 days or more	22	0.97 (0.71-1.33)	
Consumption of chicken meat (weekly)			0.328*
< 5 days	88	1	
5 days or more	24	1.15 (0.87-1.54)	
Consumption of soft drinks/ artificial juices (weekly)	95	1	0.654*
< 5 days	95	1	
5 days or more	17	1.08 (0.77-1.51)	
Consumption of sweets (weekly)			0.770*
< 5 days	86	1	
5 days or more	26	0.96 (0.71-1.29)	
Number of meals			0.191*
< 3	27	1	
4	39	0.82 (0.61-1.12)	
> 5	45	0.76 (0.56-1.03)	
Place of meals			0.658*
Home	50	1	
Work	55	1.04 (0.81-1.34)	
Restaurant	6	0.78 (0.41-1.47)	
Habit of eating watching television			0.105*
No	63	1	
Yes	47	0.81 (0.62-1.05)	
Consumption of visible fat on red meat			0.366*
No	74	1	
Yes	30	1.13 (0.86-1.49)	
Consumption of visible fat on chicken meat			0.608*
No	79	1	
Yes	28	1.08 (0.81-1.44)	
Habit of adding salt to food after cooking			0.043*
No	107	1	
Yes	5	0.45 (0.21-0.98)	

95%CI: 95% confidence interval; PR: prevalence ratio.

* The Wald Test for linear trend.

† The Wald Test for heterogeneity.

**Table 4:** Adjusted analysis of the association between overweight and the independent variables of workers at a university in the municipality of Criciúma, state of Santa Catarina, 2016/2017 (n = 214).

Variables	PR (95%CI)	P
Sex		< 0.001*
Men	1	
Women	0.66 (0.53-0.82)	
Age (years)		< 0.001^†^
18-27	1	
28-37	1.69 (1.14-2.49)	
38-47	2.13 (1.44-3.15)	
48-60	2.59 (1.80-3.73)	
Marital status		0.607*
Single	1	
Married	0.95 (0.71-1.27)	
Separated/divorced/widower	0.90 (0.58-1.38)	
Schooling (years)		0.050*
0-8	1	
9-11	0.88 (0.67-1.15)	
12 or more	0.73 (0.54-0.99)	
Consumption of legumes (weekly)		0.514*
< 5 days	1	
5 days or more	1.08 (0.85-1.38)	
Consumption of vegetables/legumes (weekly)		0.605*
< 5 days	1	
5 days or more	0.94 (0.73-1.20)	
Consumption of fruit (weekly)		0.977*
< 5 days	1	
5 days or more	1.00 (0.79-1.27)	
Consumption of red meat (weekly)		0.772*
< 5 days	1	
5 days or more	0.96 (0.70-1.30)	
Consumption of chicken meat (weekly)		0.043*
< 5 days	1	
5 days or more	1.31 (1.01-1.71)	
Consumption of soft drinks/ artificial juices (weekly)		0.879*
< 5 days	1	
5 days or more	1.03 (072-1.47)	
Consumption of sweets (weekly)		0.546*
< 5 days	1	
5 days or more	1.10 (0.81-1.51)	
Number of meals		0.524*
< 3	1	
4	0.87 (0.64-1.18)	
> 5	0.89 (0.68-1.18)	
Place of meals		0.525*
Home	1	
Work	1.16 (0.92-1.47)	
Restaurant	0.92 (0.52-1.65)	
Habit of eating while watching TV		0.972*
No	1	
Yes	0.99 (0.76-1.30)	
Consumption of visible fat on red meat		0.484*
No	1	
Yes	1.09 (0.85-1.41)	
Consumption of visible fat on chicken meat		0.490*
No	1	
Yes	0.89 (0.65-1.23)	
Habit of adding salt to food after cooking		0.040*
No	1	
Yes	0.51 (0.26-0.97)	

95%CI: 95% confidence interval; PR: prevalence ratio.

* Wald Test for linear trend.

† Wald test for heterogeneity

Regarding dietary variables, the consumption of chicken meat was associated with overweight, after adjusted analysis. Individuals who consumed chicken meat at least 5 days a week were more likely to be overweight when compared to those who ate it less than 5 days a week (PR: 1.31; 95%CI 1.01-1.71). Also, workers who had the habit of adding salt to food after cooked were less likely to be overweight compared to those who did not (habit: PR: 0.51; 95%CI 0.26-0.97). The other variables studied did not show an association with employees’ overweight (Table 4).

## DISCUSSION

An important and worrying finding in the present study is the fact that more than one-half of the employees were overweight. Similarly, other studies conducted with workers also found a high prevalence of overweight.^10^^,^^11^ These data corroborated the results of the research by Vigitel.^4^ In a decade, overweight increased 10 percentage points, from 43.2 to 53.8%.^12^

Overweight is responsible for bringing several health consequences.^13^^,^^14^ Pathologies and health damage associated with this nutritional condition include oxidative stress, metabolic syndrome, systemic arterial hypertension, type 2 diabetes mellitus, osteoarthritis, cardiovascular diseases, various types of cancer, and sleep apnea.^13^^,^^14^ In addition to the damage to physical health, overweight also causes psychosocial damage, such as body dissatisfaction, depression, negative self-perception, and social discrimination.^13^^,^^14^ The complications of overweight create a vicious cycle, since they lead to a reduction in physical activity and healthy behaviors, maintaining the pattern of overweight and health damage.^14^

The aforementioned studies assessed overweight using BMI ≥ 25 kg/m^2^, which allowed comparability with the data in the present study. At the population level, the most widely used measure of nutritional status classification is BMI. Its result is obtained by dividing weight into kilograms by height in squared meters, with individuals with BMI ≥ 25 kg/m^2^ classified as overweight.^1^ It is necessary to emphasize that the BMI has some limitations, such as not being able to differentiate muscle mass from body fat and not clearly assessing the distribution of adipose tissue.^1^

However, to assess nutritional status in population studies, the use of self-reported weight and height has become a fast and low-cost tool.^15^^,^^16^ According to Thomaz et al.,^16^ 93% of men and 97% of women who are overweight, as well as 92% of men and 93% of eutrophic women, correctly report their weight and height data. Thus, using these data offers results very close to the measurement,^15^ allowing its use in studies that seek to identify the nutritional status of the population.^16^

According to some authors, men with low weight tend to overestimate their weight, while obese people tend to underestimate it.^15^^,^^16^ Women often underestimate weight information; however, those with low weight tend to overestimate it.^15^ Height is also usually overestimated in both sexes.^15^ However, such differences between the measured and reported values are not significant.^15^^,^^16^

Another result evidenced in this study was that, even after adjusting for possible confounding factors, women maintained a 34% lower risk of overweight compared to men. Similarly, other studies also found a higher risk of overweight among men.^17^^,^^18^ Gonçalves et al.^17^ showed that men were twice as likely to be overweight as women. Similarly, another study showed that 63.6% of men and 49.7% of women working at a university were overweight.^18^

Women generally have body standards to follow and nutritional status is responsible for affecting their level of body satisfaction.^19^ For them, body appearance, in most cases, is more important than their nutritional status.^19^ According to some authors, women who are overweight are more likely to be dissatisfied with their body image.^20^ Thus, this concern makes them seek a healthier lifestyle, which is represented in a study by Pretto et al.,^21^ who found that women had a “healthier profile” when compared to men because they consumed more fruit and vegetables, did not usually add salt to the food after cooking, had a greater tendency to perform physical activity, in addition to having an adequate BMI.^21^

Regarding the association between age and overweight, a direct relationship between the variables was observed. Other studies have also verified this result.^8^^,^^22^ Siqueira et al.^8^ found that individuals in a higher age group had a higher prevalence of overweight and obesity.^8^ A cohort study conducted in the municipality of Pelotas, in the state of Rio Grande do Sul, also found an increase in body weight with increased age,^22^ showing that, from 15 to 30 years of age, the prevalence of overweight went from 23.2 to 57.6%.^22^

A possible explanation for this finding is that, along with aging, there is a decrease in the muscle mass of the human organism and, at the same time, an increase in body fat.^23^ Adipose tissue has been considered a component of great importance for immunological, endocrine, and metabolic processes; therefore, it becomes responsible for affecting the balance of the entire human body.^24^ With the development of excess body fat, there is an imbalance in the functioning of the adipose tissue, leading to an inflammatory process.^24^ This mechanism does not only affect adipose tissue, but several other main organs for the performance of metabolism,^24^ leading to the development of dyslipidemia, insulin resistance, and, as a consequence, type 2 diabetes mellitus, besides non-alcoholic liver steatosis, and bone fragility.^25^

In the present study, an inverse relationship between schooling and overweight was evidenced. Similarly, a survey conducted with university employees showed that the prevalence of overweight among those who had up to 12 years of schooling was 67.1%, while employees with more than 12 years of schooling had a prevalence of 45.3%.^17^ Another study also showed a higher prevalence of overweight (40.5%) and obesity (27.8%) among workers who had studied until elementary school compared to those who studied up to high school or higher education.^8^

According to Ng,^26^ people with higher levels of schooling tend to belong to the highest socioeconomic level and, therefore, have greater knowledge about healthy eating. Thus, it can be highlighted that stimulating health education in countries with lower socioeconomic conditions could increase the consumption of nutritionally balanced meals and access to knowledge about health and, consequently, prevent overweight.^26^

The dietary variables that were related to the employees’ overweight were consumption of chicken meat at least 5 days a week and not adding salt to the food after it was cooked. In contrast, research has shown that chicken intake was not associated with abdominal obesity in men or generalized obesity in both sexes.^27^ Also, the study by Ma et al.^28^ found that salt intake is higher among overweight individuals. A possible explanation for the finding of the present study refers to the reverse causality, commonly found in cross-sectional studies. That is, because they are overweight, employees may be controlling excess salt. According to Silva,^29^ individuals who are overweight, have high blood pressure, and diabetes usually receive more dietary guidelines in relation to the consumption of salt, fat, and sugar. The other independent variables showed no association with the excess weight of the employees evaluated in this study, which corroborates the results of other studies.^8^^,^^30^

It is important to highlight some limitations of this study. The lack of information on workers’ income did not allow to evaluate the association between this exposure variable and overweight. However, in this study, the education variable was evaluated and used as a proxy. As a strong point, we highlight the pioneering nature of this study at the university where it was performed, since it is the first study developed with the employees of this institution, the only university in a region that covers about 1 million people. The methodological rigor, the standardization and training of the interviewers, and the double typing used in this work also stand out.

## CONCLUSIONS

The current results have allowed us to evaluate the factors related to overweight in university employees for the first time. Men and older individuals showed the highest prevalence of overweight. Also, employees with higher levels of schooling were less likely to be overweight.

Considering that more than one-half of the institution’s workers were overweight, it is essential and urgent to develop interventions and / or programs that promote health in the work environment, especially aimed at higher risk groups, such as older men and those with less schooling.

It is also necessary to encourage research focused on workers’ health, which is an area still little explored, although it is highly relevant to public health.
